# Improved SNR and Image Quality of Pediatric MRI with a Shape Conforming High-Density Posterior Cushion Coil

**DOI:** 10.3390/bioengineering13080881

**Published:** 2026-07-30

**Authors:** Terrence Jao, Praveen Jayapal, Jana Vincent, John Pauly, Ceara Stack, Victor Taracila, Fraser Robb, Thomas Grafendorfer, Yun Jeong Stickle, Mark Giancola, Clyve Konrad Follante, Greig Scott, Shreyas Vasanawala, Ali B. Syed

**Affiliations:** 1 Department of Radiology, Stanford University, 300 Pasteur Ave., Stanford, CA 94305, USAvasanawala@stanford.edu (S.V.);; 2 General Electric Healthcare, Aurora, OH 44202, USA; jana.vincent@gehealthcare.com (J.V.);; 3 Department of Electrical Engineering, Stanford University, Stanford, CA 94305, USA

**Keywords:** pediatric MRI, radiofrequency coil array, high density coil, conformal coil, signal-to-noise ratio, image quality, pediatric abdominal MRI

## Abstract

A high-density posterior cushion coil (HDPCC) can improve the SNR in pediatric MRI by conforming to the body and minimizing distance to the imaged volume when compared to a conventional rigid built-in table coil array. Posterior coil arrays built into MRI tables are mainstays but provide suboptimal performance in pediatric imaging due to their distance from the imaged volume and lower coil density. Our goal is to improve pediatric MRI by constructing a prototype 30-channel high-density posterior cushion coil (HDPCC) incorporated into a padded cushion. Twenty-five pediatric patients were scanned with the HDPCC, and twenty-five pediatric controls served as the comparison group using standard coils. Two radiologists graded image quality on a 5-point scale. SNR was measured in the right paraspinal musculature and the liver. All images obtained with the HDPCC were diagnostic (mean score 4.13/5). SNR in the superficial tissues of the paraspinal musculature was 34% higher with the HDPCC than with standard coils, while SNR in the deeper tissues of the liver was statistically similar between the two coils. HDPCC yields improved the signal-to-noise ratio in phantoms and pediatric study participants by conforming to the body.

## 1. Introduction

Pediatric MRI faces unique challenges due to the mismatch between adult-sized, rigid commercial coils and the smaller body habitus of children [1,2,3]. Traditional posterior coil arrays are often built into MRI tables, underneath cushions and plastic, which increases the distance to the patient and limits the maximal achievable SNR. Additionally, the rigid elements cannot conform to the smaller body sizes of pediatric subjects, which reduces the number of effective coil elements. Much work has been undertaken to design conformal pediatric coils to maintain SNR, including small head coils for infants and toddlers [4,5,6,7,8,9,10,11,12,13], flexible coils that conform to the patient [14,15,16,17,18,19,20], as well as screen-printed coils that can be printed on fabric [21,22,23]. Recently, a dense 60-channel flexible blanket-type small loop array with a novel dual turn loop configuration has shown superior parallel imaging performance compared with standard coils [24].

In this paper, we assess a prototype, high-density posterior cushion coil (HDPCC), which is integrated into a padded cushion to promote patient comfort while conforming tightly and closely to the relevant anatomy. The thickness of the cushion additionally provides the ability to position smaller patients nearer to the magnet isocenter. We seek to characterize the image quality and SNR in clinical practice compared to standard-of-care coils.

## 2. Material and Methods

Several authors (Jana Vincent, Fraser Robb, Thomas Grafendorfer, Yun Jeong Stickle, Mark Giancola, and Clyve Konrad Follante) are employees of G.E. Healthcare. G.E. Healthcare also gave financial support for this study. The authors who are not employees of G.E. Healthcare had control over data inclusion that may present any conflict of interest for the aforementioned authors.

**Figure 1:**
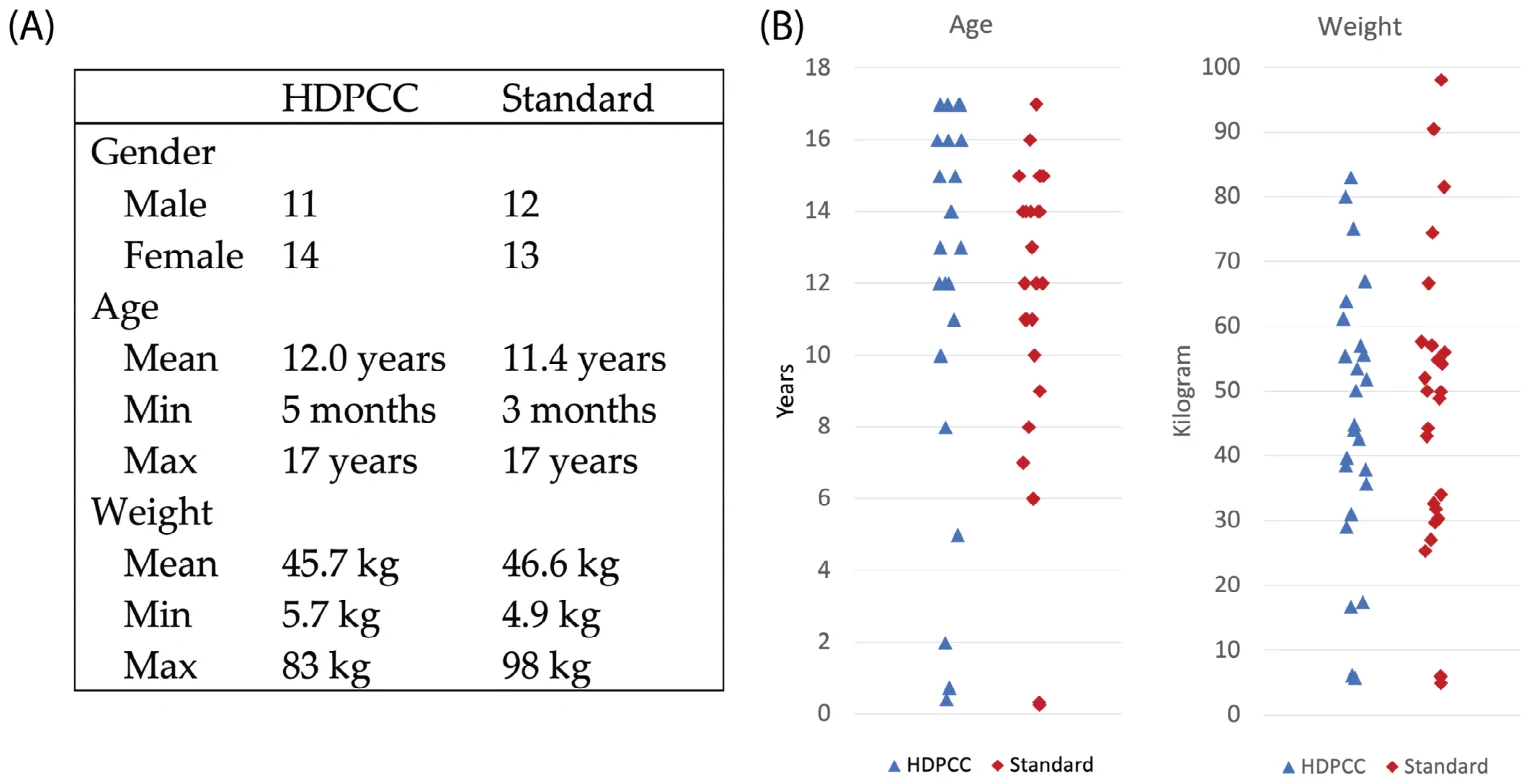
Patient demographics. (**A**) A total of 47 patients were retrospectively selected for this study with 2 patients that were scanned on both coils. (**B**) Dot plots of the patient age and weight are shown using the HDPCC (blue triangle) and standard coil (red diamond).

This study was approved by an institutional review board and informed consent was obtained. Forty-seven pediatric patients (50 studies) referred for clinically indicated abdominal and/or pelvis MRI exams were scanned using a 3T GE Signa Architect (Chicago, IL, USA). Twenty-five studies were scanned with the HDPCC after providing informed consent/assent between December 2019 and October 2020. A control group was also selected of pediatric patients referred for 3T MRI during the same timeframe on the same scanner, using the standard 40-channel posterior array built into the scanner table. The control group was selected to be age- and weight-matched to ensure similar patient size and distribution between the groups. Two participants were scanned with both coils in different sessions. In these two patients, the examinations were performed on average of 5.5 months apart as part of routine clinical surveillance. Subjects age ranged from 2 months to 17 years with further demographic details included in Figure 1.

All examinations were performed as part of the routine clinical workflow by independent imaging technologists. While baseline acquisition parameters were bounded by the institutional pediatric imaging protocols, technologists could make patient-specific adjustments solely to ensure adequate diagnostic quality. These adjustments are limited to routine modifications, such as tailoring the field-of-view and scan coverage to account for patient size. Core parameters including the repetition time (TR), echo time (TE), receiver bandwidth (BW), and flip angle (FA) were not manually adjusted. They were set by the protocol and could be dynamically modified by the pulse sequence software based on the prescribed coverage. Detailed scan parameters are provided in Table 1. None of the study authors had any control over coil assignment or scan parameters.

## 3. Coil Design and Construction

**Figure 2:**
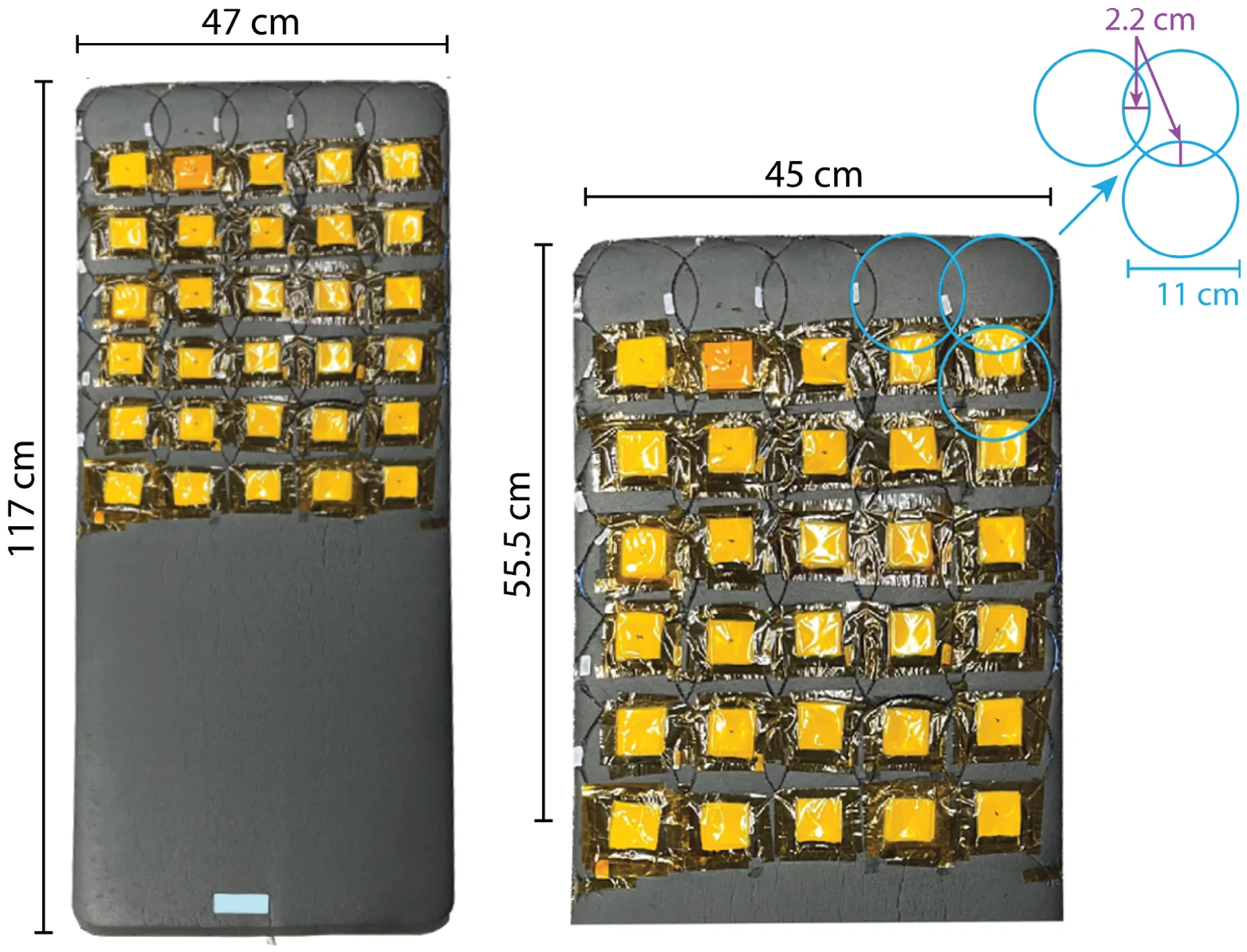
The HDPCC coil is a 30-channel flexible array with 11 cm coils with 2.2 cm (20%) of critical overlap arranged on a 5 × 6 grid. The overall dimension of the HDPCC coil measures 47 × 117 cm with the coil elements measuring 45 × 55.5 cm.

The HDPCC is a 30-channel flexible array with an element size of 11 cm and critical overlap of 2.2 cm (20%) (Figure 2). These elements and cabling were embedded into the top, patient side of a conformal foam pad. This provided the stability of the elements while allowing natural contouring of the elements as closely as possible to the pediatric subjects. The foam density was chosen such that the lighter weights of the pediatric populations could deform the foam for the closest fit while allowing mechanical support for the element and elevation of the pediatric subjects closer to isocenter. Coil elements have an electronics module comprised of a preamplifier, active and passive decoupling, matching and tuning circuitry, and balun. The finished coil was covered with a biocompatible, cleanable sleeve.

The HDPCC was compared against a 40-channel built-in table posterior array (element size 9 × 3 cm to 10 × 9 cm), which was used in the control cohort. The anterior coil was standardized between the two groups. A lightweight and flexible 30-channel anterior AIR coil (element size 14.4 cm) was used in combination with the posterior coils across both cohorts. Due to the flexible blanket-like design of the anterior coil, this single coil conformed to varying body sizes, except in two infants less than 6 months old, who were wrapped in a 16-channel GEM Flex Medium coil (element size 8 cm) along the anterior body.

## 4. SNR and Image Quality

### 4.1. Phantom SNR

SNR was measured in a rectangular phantom at ROIs of varying depths both centered on the coil element and at the area of maximal overlap between coil elements.

### 4.2. Image SNR

Image SNR was calculated on a subset of examinations (12 HDPCC, 12 standard) that included a multi-echo gradient echo T2* mapping sequence using a receiver bandwidth of 62.5 kHz and TR/TE of 30.4 ms/0.94 ms. The two infants scanned with the GEM Flex coil were excluded from quantitative SNR analysis to maintain consistent coil configuration between groups. This sequence was chosen for SNR measurements because it did not use any vendor-specific acceleration, filtering, or surface intensity correction. For most patients, a baseline FOV and slice thickness of 320 mm and 8 mm was appropriate, resulting in a baseline voxel size of 3.33 × 3.33 × 8 mm with an acquisition matrix of 96 × 96. In three patients, a different FOV was used, leading to a different voxel size. In these patients, the SNR was normalized to the baseline voxel size as follows:

$${\mathrm{SNR}}_{\mathrm{normalized}}={\mathrm{SNR}}_{\mathrm{raw}}\times \left(\frac{V_{\mathrm{baseline}}}{V_{\mathrm{acquired}}}\right)$$

Additionally, in two subjects, the T2* mapping sequence was acquired with two signal averages. In these two patients, the SNR was normalized by dividing by $\sqrt{2}$ to match the single-average equivalent. SNR was measured as the mean signal value within a region of interest (ROI) of the right paraspinal musculature and segment two of left hepatic lobe divided by the background noise standard deviation [25,26]. The noise standard deviation was calculated over a non-signal-producing background ROI, as raw noise-only reconstructions were not available from the clinical workflow. The independent *t*-test was used to evaluate the statistical difference in SNR between the HDPCC and the standard coil, while Cohen’s *d* was used to evaluate the effect size, where thresholds of 0.2, 0.5, and 0.8 represent small, medium, and large effects.

### 4.3. Image Quality

**Figure 3:**
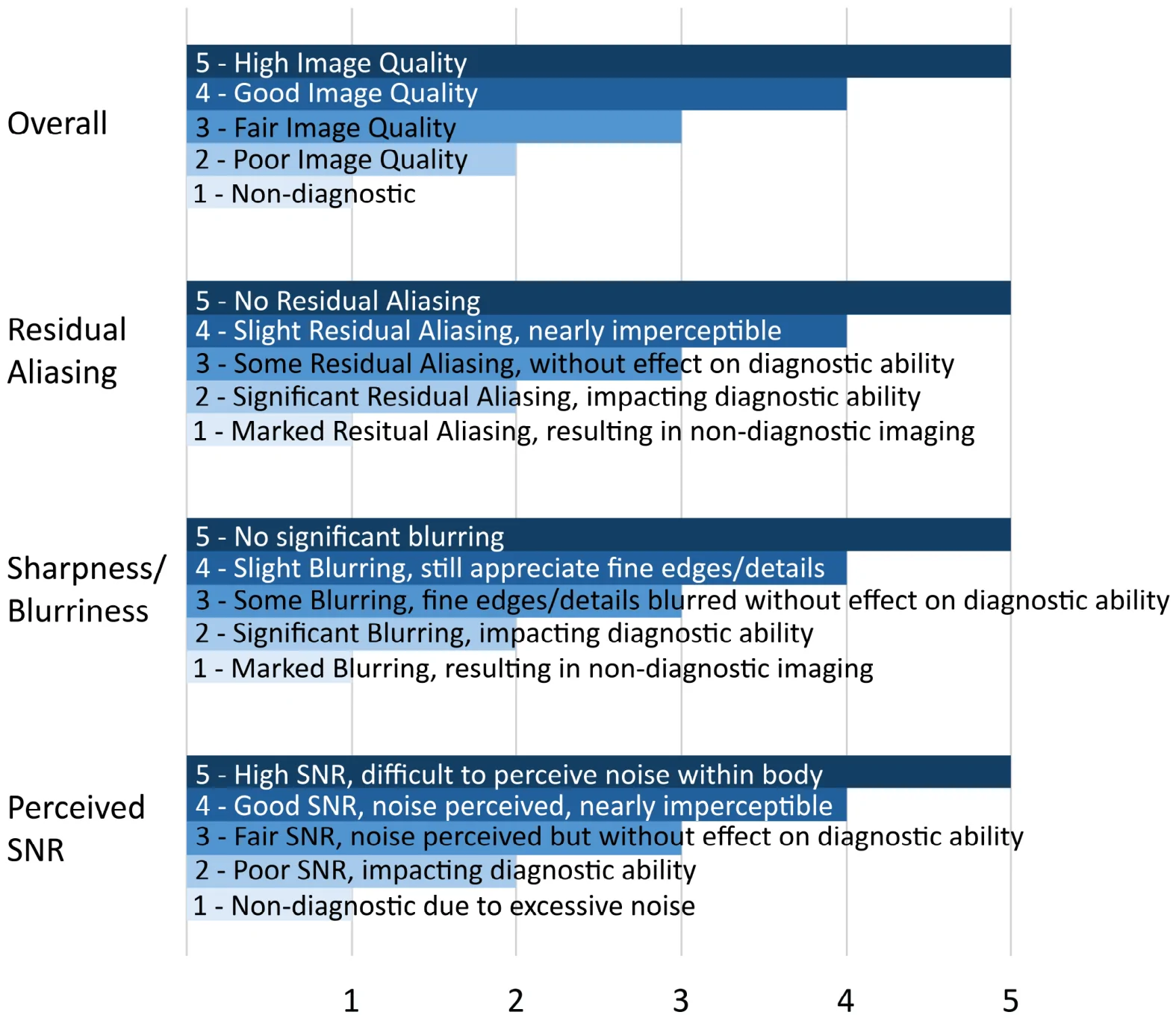
Likert-scale grading was performed by two board-certified radiologists on a scale from 1 to 5 in terms of overall image quality, residual aliasing, sharpness/blurriness, and perceived SNR.

Two board-certified pediatric radiologists (10 and 8 years of experience) rated the image quality for each examination on a coronal T2-weighted single-shot fast spin echo (Cor SSFSE) with acceleration factors ranging from 2 to 3 and a post-contrast axial T1-weighted fast spoiled gradient-echo Dixon sequence (Ax T1 FSPGR) using the in-phase echo with acceleration factors ranging from 2 to 2.5. The detailed scan parameters are in Table 1. To ensure rigorous blinding, patient images were anonymized on PACS to remove all metadata identifying coil selection. HDPCC and standard coil studies were completely intermixed and presented in randomized batches to two radiologists who evaluated the images independently. Images were graded on a 5-point Likert scale in terms of perceived SNR, sharpness and blurring, residual aliasing, and overall diagnostic image quality (Figure 3). A Mann–Whitney test with Holm–Bonferroni correction for multiple comparisons was used to evaluate the statistical difference between the HDPCC and the standard coil. Effect size was measured using the non-parametric rank-biserial correlation coefficient *r*, calculated as the z-score divided by the square root of the total sample size ($\left|Z\right|/\sqrt{N}$), where thresholds of 0.1, 0.3 and 0.5 denote small, medium, and large effect sizes. Confidence intervals at the 95% level were estimated non-parametrically through bootstrapping. The data were resampled with replacement, and the lower and upper bounds were determined from the 2.5th and 97.5th percentiles of the bootstrap distribution. Inter-reader agreement for the Likert-scale grades was evaluated using quadratic-weighted Gwet’s AC2 coefficients, as standard Cohen’s kappa statistics can be artificially depressed under high-agreement, low-variance ceiling conditions [27].

## 5. Results

### 5.1. Phantom SNR

**Figure 4:**
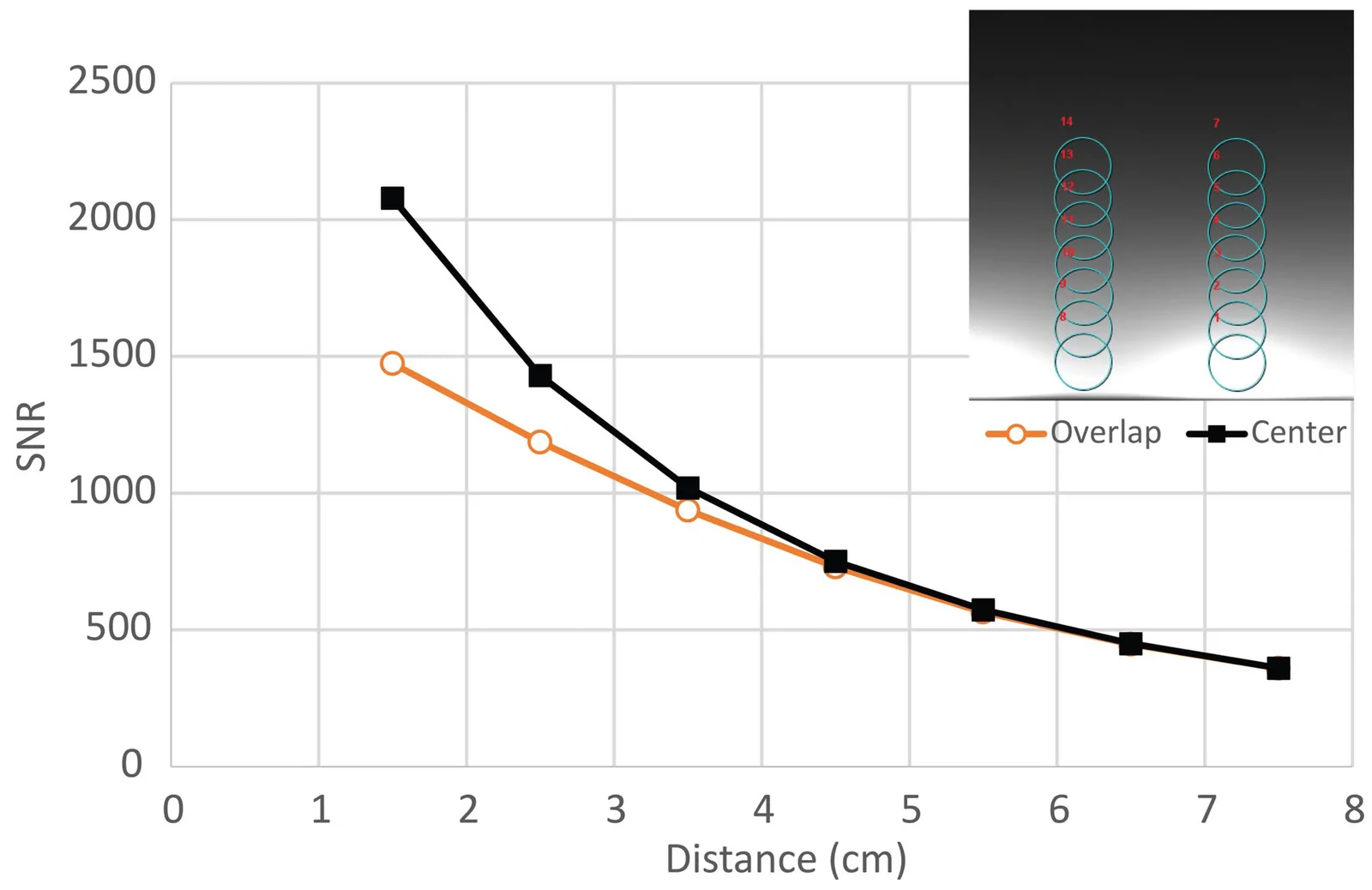
SNR calculated at various depths in a phantom both centered (black square) and at maximal overlap (orange circle) of the coil elements. Insert of the phantom and ROIs are shown in the top right corner.

Figure 4 displays the SNR at various depths in a phantom. SNR decreased with increased depth and is increased when centered on the coil elements. The coil covariance matrix demonstrates relatively low noise correlation between coil elements.

### 5.2. Image SNR

**Figure 5:**
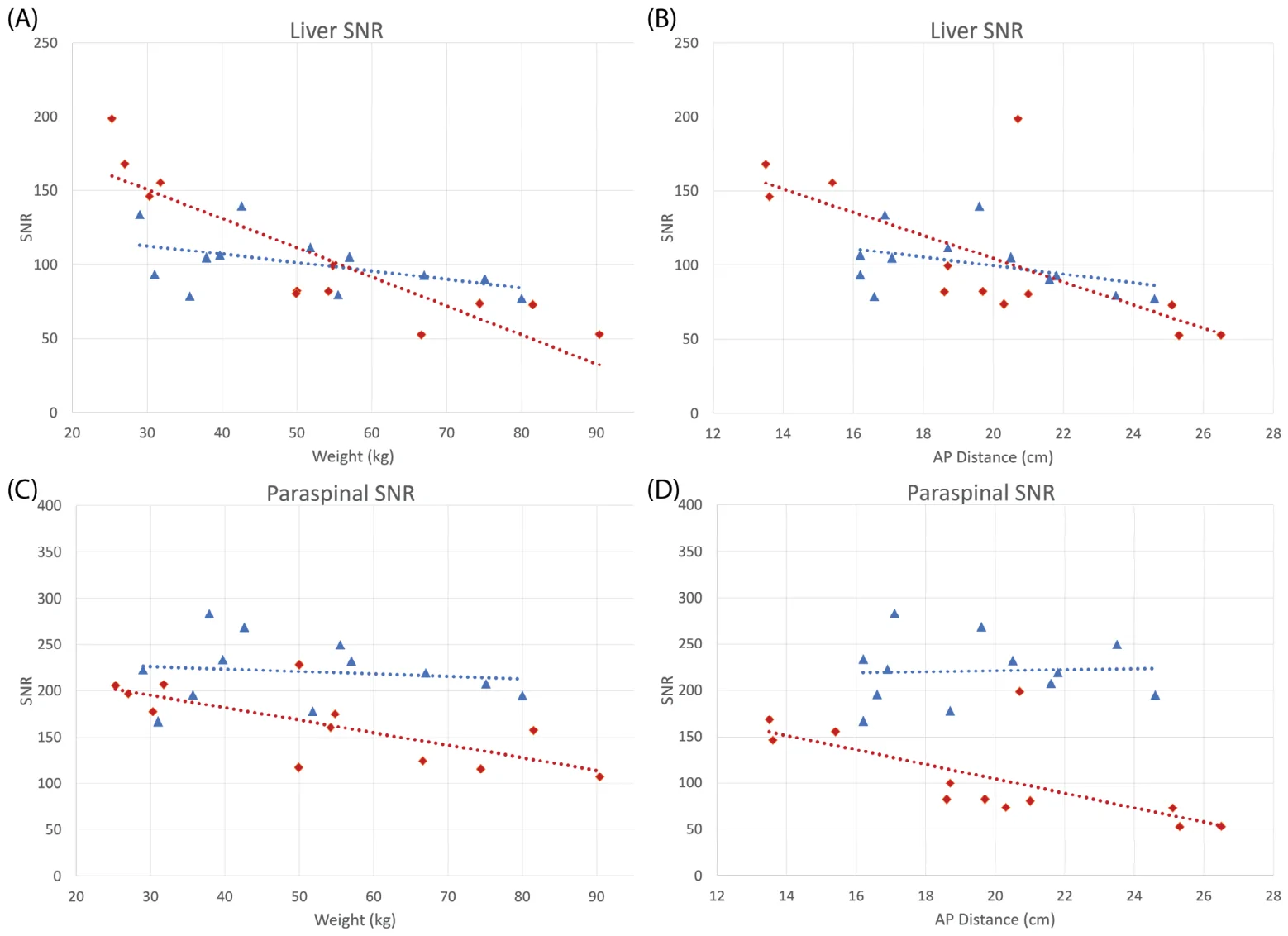
SNR within the (**A**) liver and (**C**) paraspinal musculature versus patient weight as well as the (**B**) liver and (**D**) paraspinal musculature versus anterior–posterior diameter using the HDPCC (blue triangle) and standard coil (red diamond).

The mean image SNR of the HDPCC was 221 ± 35 (paraspinal) and 101 ± 20 (liver) compared to 164 ± 41 and 105 ± 49, respectively, using the standard coil. The HDPCC coil had higher SNR in the paraspinal musculature (mean difference: 57, 95% CI: 25–89, Cohen’s *d* = 1.49, *p* = 0.01) and similar SNR in the liver (mean difference: −4, 95% CI: −37–28, Cohen’s *d* = −0.11, *p* = 0.78). Figure 5 demonstrates SNR as a function of both patient weight and the anterior–posterior diameter in both the liver and paraspinal musculature.

### 5.3. Image Quality

**Figure 6:**
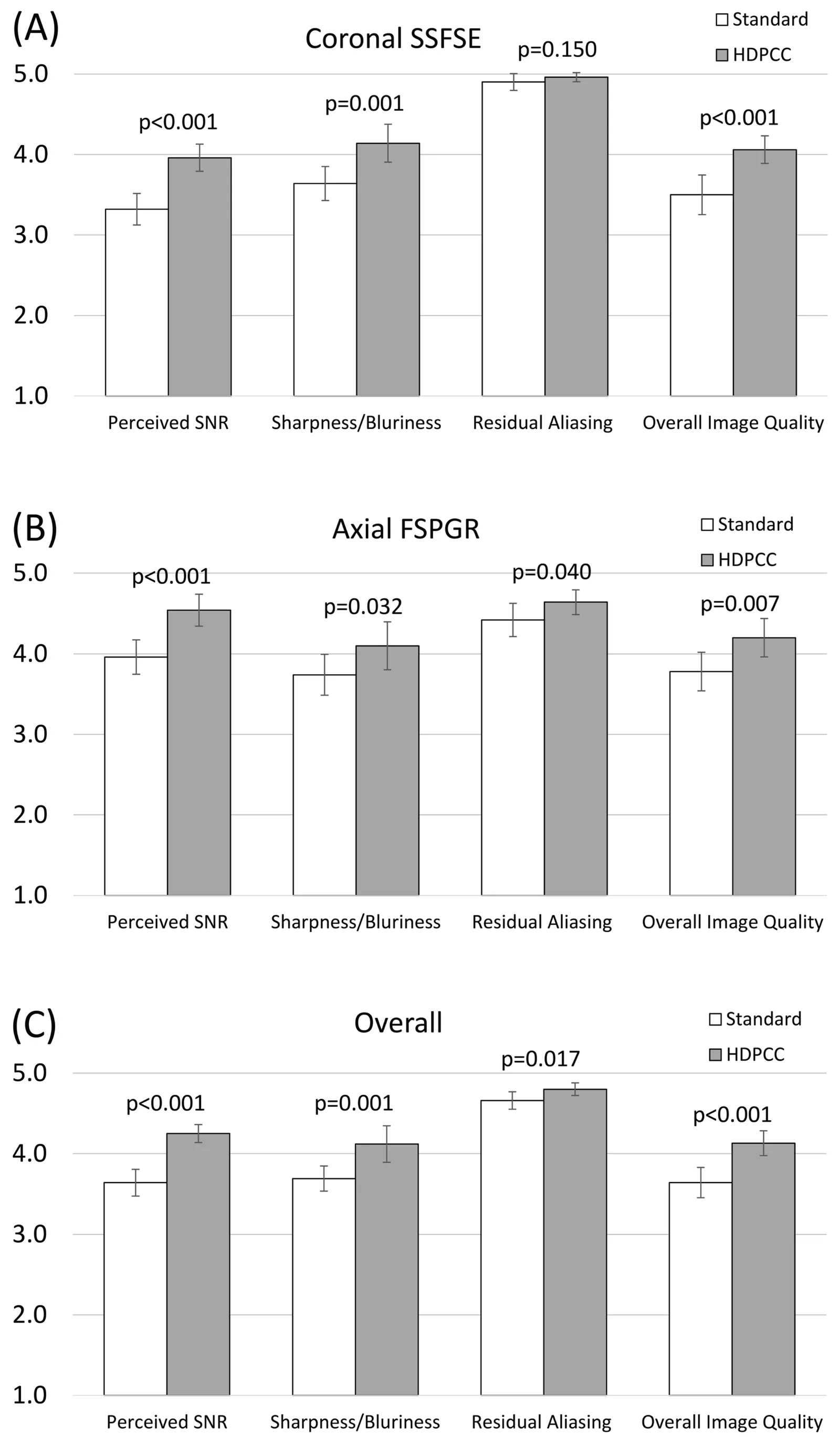
Image quality grading assessment in terms of perceived SNR, sharpness/blurriness, residual aliasing, and overall image quality on a 5-point Likert scale (5 = high image quality, 4 = good image quality, 3 = fair image quality, 2 = poor image quality, 1 = nondiagnostic). Grading was assessed on (**A**) coronal T2-weighted SSFSE, (**B**) axial T1-weighted FSPGR post-contrast, and (**C**) overall (average between SSFSE and FSPGR).

**Figure 7:**
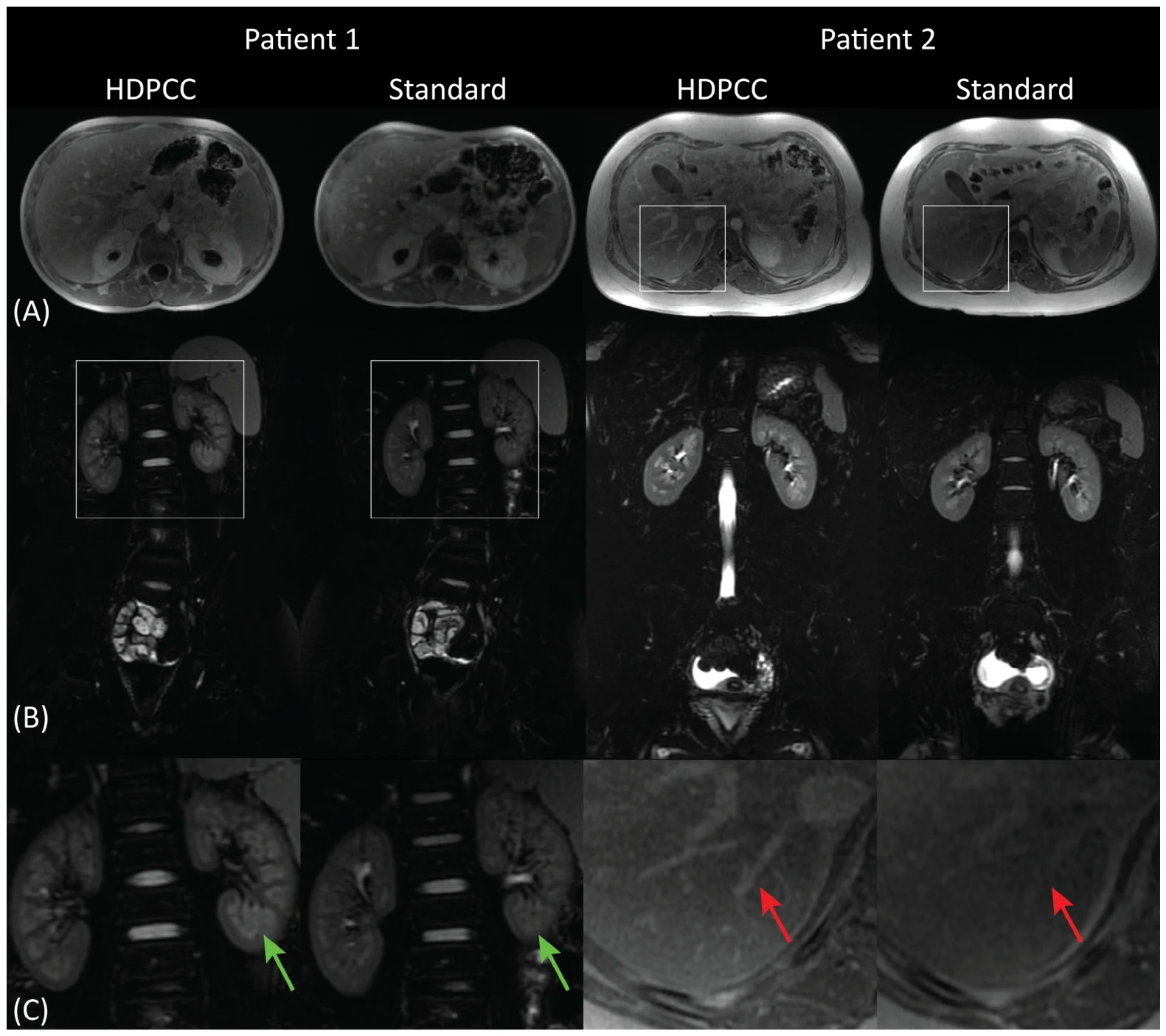
Representative images of (**A**) Ax FSPGR in-phase echo and (**B**) Cor SSFSE used for image quality grading in two patients that were scanned using both the HDPCC and the standard coil. (**C**) Magnified views of the kidneys and the posterior liver are demarcated by a white box in the full-field images. Qualitatively, there is increased differentiation of the renal medulla and renal cortex on Cor SSFSE (green arrow) and the hepatic vasculature on Ax FSPGR (red arrow) when using the HDPCC. Specific scan parameters (TR [ms]/TE [ms]/FOV [mm]/BW [Hz]) were as follows: Patient 1: Coronal SSFSE: 991/153/360/325 (HDPCC) and 976/154/360/325 (Standard); Axial FSPGR: 6.6/2.5/320/650 (HDPCC) and 6.4/2.3/300/650 (Standard). Patient 2: Coronal SSFSE: 920/178/460/325 (HDPCC) and 838/172/460/325 (Standard); Axial FSPGR: 5.8/2.2/400/650 (HDPCC) and 4.6/2.2/440/650 (Standard).

All 25 examinations using the HDPCC were diagnostically acceptable with an overall image quality score of 4.13 ± 0.38 (95% CI 3.98–4.27), which was significantly higher than the overall image quality of the 25 examinations using the standard coil with a score of 3.64 ± 0.46 (95% CI 3.47–3.82), leading to an effect size of *r* = 0.59 and *p*-value < 0.001. The perceived SNR, sharpness/blurring, and residual artifacts also scored higher with the HDPCC when compared with the standard coil and are detailed in Figure 6. The overall image quality grading by the two radiologists demonstrated substantial inter-reader agreement with quadratic-weighted Gwet’s AC2 values of 0.86 (95% CI 0.79–0.92) for the Cor SSFSE sequence and 0.79 (95% CI 0.71–0.88) for the Ax FSPGR (LAVA) sequence. Two subjects were scanned with both the HDPCC and the standard coil, and representative images are shown in Figure 7.

## 6. Discussion

In this paper, we compared an HDPCC with a conventional, standard-of-care posterior coil and demonstrate a higher perceived SNR, sharpness, and overall image quality of the HDPCC over the standard coil with high inter-reader agreement between two board-certified pediatric radiologists. We also measured the SNR in vivo and showed statistically higher SNR in the more superficial tissues of the paraspinal musculature with the HDPCC with large corresponding effect sizes, while deeper tissue of the liver showed similar SNR values between coils. This is an expected result, given that superficial tissues are closer to the posterior array while deeper tissues are further away, where there is reduced coil sensitivity at greater depth.

However, we did notice that the SNR trendline suggests a slightly higher liver SNR in the control group than in the HDPCC group for the smallest patients in terms of weight and anterior–posterior diameter (Figure 5A,B). We believe this is due to a mismatch in the cohort distribution rather than a true performance limitation of the HDPCC. The control cohort includes two subjects that weigh less than 27 kg and three subjects with anterior–posterior diameter less than 15 cm who are absent from the HDPCC cohort due to re-world clinical scheduling variations. In these smaller patients, a deep anterior structure like the liver rests closer to the posterior coils, artificially increasing the SNR baseline of the control trendline. Meanwhile, within the overlapping spectrum where patient sizes are equivalent between the cohorts, the HDPCC demonstrates equivalent performance with the control cohort.

This paper has a few limitations. Scan parameters were allowed to vary at the technologist’s discretion to obtain images of diagnostic quality within an acceptable scan time in a busy clinical workflow, and these are detailed in Table 1. While this variability may introduce potential bias in grading images, analysis of key parameters including TR, TE, matrix size, and slice thickness showed no significant differences between the control and HDPCC cohorts (all parameters *p* > 0.35). Therefore, parameter variability is unlikely to have introduced systematic bias in image quality scoring. We did not statistically compare the same subject with both the HDPCC and standard coil, as this would be infeasible in patients receiving general anesthesia, although we were able to show a few illustrative examples in Figure 7.

While we demonstrated improved SNR in superficial anatomy, our image quality assessment was limited to MRI exams of the abdomen and/or pelvis. Imaging studies that emphasize more superficial anatomy, given their closer proximity to the posterior array, could benefit more greatly from the HDPCC, such as spine imaging. Additionally, our analysis only focused on image quality and not on pathology detection. Future work can also focus on comparing the parallel imaging performance of the HDPCC with conventional coils. The high-density 30-channel layout used in the HDPCC was designed to provide highly distinct spatial sensitivity profiles, which are necessary to minimize parallel imaging noise amplification during high acceleration factors [28,29,30,31]. However, a systematic comparison of maximum acceleration thresholds was outside the scope of this clinical evaluation.

## 7. Conclusions

We demonstrate that a conformal, high-density posterior cushion coil (HDPCC) yielded higher perceived diagnostic image quality with SNR that is higher in superficial tissues and similar in deeper tissues when compared with a standard coil.

## Figures and Tables

**Table 1:** Scan parameters are presented as the mean value with standard deviation in parentheses. Parameters were adjusted at the discretion of the technologist to obtain diagnostic quality images in an acceptable scan time, resulting in variations across individual examinations. However, there were no statistically significant differences in acquisition parameters between the HDPCC and control cohorts for either the Coronal SSFSE or Axial FSPGR sequences (all *p* > 0.35, two-sample unpaired *t*-test). The T2* mapping sequence had fixed scan parameters for all patients.

	Coronal SSFSE		Axial FSPGR		T2* Mapping
	HDPCC	Control	HDPCC	Control	
TR (ms)	1147 (499)	1212 (506)	7.3 (1.0)	7.2 (1.2)	30.4
TE (ms)	151 (36)	143 (34)	2.4 (0.1)	2.4 (0.1)	0.94
BW (Hz)	325	325	650	650	650
Matrix	322 × 227 (33 × 19)	316 × 224 (22 × 17)	308 × 251 (23 × 38)	298 × 258 (30 × 31)	96 × 96
Slice Thick (mm)	3.9 (0.8)	4.0 (0.5)	2.3 (0.3)	2.4 (0.3)	8
Acquisition Time (sec)	66 (44)	65 (37)	38 (33)	45 (55)	2.3

## Data Availability

To comply with the Health Insurance Portability and Accountability Act (HIPAA), the patient imaging datasets evaluated in this study contain protected health information (PHI) and cannot be made publicly available.
